# Establishment of an integrated model incorporating standardised uptake value and N-classification for predicting metastasis in nasopharyngeal carcinoma

**DOI:** 10.18632/oncotarget.7253

**Published:** 2016-02-08

**Authors:** Yuan Zhang, Wen-Fei Li, Yan-Ping Mao, Guan-Qun Zhou, Hao Peng, Ying Sun, Qing Liu, Lei Chen, Jun Ma

**Affiliations:** ^1^ Department of Radiation Oncology, Sun Yat-sen University Cancer Center, State Key Laboratory of Oncology in South China, Collaborative Innovation Center for Cancer Medicine, Canton, Guangdong, China; ^2^ Department of Medical Statistics and Epidemiology, School of Public Health, Sun Yat-sen University, Canton, Guangdong, China

**Keywords:** nasopharyngeal neoplasms, metastasis, TNM staging, maximum standardized uptake value, recursive partitioning analysis

## Abstract

**Background:**

Previous studies reported a correlation between the maximum standardised uptake value (SUV_max_) obtained by ^18^F-fluorodeoxyglucose (^18^F-FDG) positron emission tomography (PET) and distant metastasis in nasopharyngeal carcinoma (NPC). However, an integrated model incorporating SUV_max_ and anatomic staging for stratifying metastasis risk has not been reported.

**Results:**

The median SUV_max_ for primary tumour (SUV-T) and cervical lymph nodes (SUV-N) was 13.6 (range, 2.2 to 39.3) and 8.4 (range, 2.6 to 40.9), respectively. SUV-T (HR, 3.396; 95% CI, 1.451-7.947; *P* = 0.005), SUV-N (HR, 2.688; 95%CI, 1.250-5.781; *P* = 0.011) and N-classification (HR, 2.570; 95%CI, 1.422-4.579; *P* = 0.001) were identified as independent predictors for DMFS from multivariate analysis. Three valid risk groups were derived by RPA: low risk (N0-1 + SUV-T <10.45), medium risk (N0-1 + SUV-T >10.45) and high risk (N2-3). The three risk groups contained 100 (22.3%), 226 (50.3%), and 123 (27.4%) patients, respectively, with corresponding 3-year DMFS rates of 99.0%, 91.5%, and 77.5% (*P* <0.001). Moreover, multivariate analysis confirmed the RPA-based prognostic grouping as the only significant prognostic indicator for DMFS (HR, 3.090; 95%CI, 1.975-4.835; *P* <0.001).

**Methods:**

Data from 449 patients with with histologically-confirmed, stage I-IVB NPC treated with radiotherapy or chemoradiotherapy were retrospectively analysed. A prognostic model for distant metastasis-free survival (DMFS) was derived by recursive partitioning analysis (RPA) combining independent predictors identified by multivariate analysis.

**Conclusion:**

SUV-T, SUV-N and N-classification were identified as independent predictors for DMFS. An integrated RPA-based prognostic model for DMFS incorporating SUV-N and N-classification was proposed.

## INTRODUCTION

Nasopharyngeal carcinoma (NPC) is particularly prevalent in southern China, Southeast Asia, North Africa, the Middle East, and Alaska [1]. Radiotherapy is the primary treatment used for non-disseminated NPC [2, 3]. With advances in imaging and radiation therapies, local-regional control has exceeded 90% [4]. However, 20-30% of NPC patients eventually develop distant metastasis [5–8], which accounts for the majority of failures [7, 8]. Effort should therefore be made to stratify patients into different groups based on the risk of metastasis to tailor individualized treatments and improve outcomes.

N-classification in the TNM staging system is a measure of the extent of node involvement, and is currently the most reliable tool for assessing metastasis risk in NPC [9, 10]. However, there is remaining room for improvement in the correlation between the N classification and metastasis [11, 12], perhaps because N-classification is based solely on anatomic extent and lacks non-anatomic information such as tumour physiology.

^18^F-fluorodeoxyglucose (^18^F-FDG) positron emission tomography (PET) imaging is used to probe glucose metabolism in tumour cells [13]. The maximal intensity of FDG uptake by the tumour (maximum standardized uptake value; SUV_max_) is a valuable marker of tumour biological behaviour [13, 14] and a useful predictor of distant metastasis in NPC [15, 16]. However, an integrated model incorporating SUV_max_ and anatomic staging for stratifying metastasis risk has not been reported. Clinicians are therefore somewhat troubled as to how best to incorporate SUV_max_ into clinical decision-making. A valid approach for incorporating non-anatomic prognostic factors and anatomic staging into an integrated prognosis grouping was recently described [17], which significantly improved survival prediction compared with previous models. In the present study, we extended this approach by using recursive partitioning analysis (RPA) to develop an integrated prognostic model for metastasis that combines SUV parameters and N-classification.

## RESULTS

### Treatment failure and survival

The median follow-up time was 49.5 months (range, 3.37–67.9 months), and 385/402 (95.8%) of surviving patients were followed up for >3 years. A total of 84 patients experienced treatment failure, with 19/449 (4.2%), 21/449 (4.7%), and 53/449 (11.8%) developing local recurrence, regional recurrence, and distant metastases, respectively. 8/449 (1.8%) patients experienced both local-regional recurrence and distant metastases and 47/449 (10.5%) patients died- the causes of death were nasopharyngeal carcinoma (93.6%, 44/47), other diseases (2.1%, 1/47) and unknown causes (4.3%, 2/47). The 3-year distant metastasis-free survival, local relapse-free survival, regional relapse-free survival, disease-free survival and overall survival was 89.4%, 96.6%, 95.6%, 83.9% and 94.4%, respectively.

### Prognosis of different N subcategories

In total, 75 (16.7%), 251 (55.9%), 76 (16.9%), and 47 (10.5%) patients were classified as N0-3, respectively. The 3-year DMFS decreased only very slightly with increasingly higher N category (96.0%, 93.2%, 81.1% and 71.7%, *P* <0.001). However, no significant differences were observed between N0 and N1 (*P* = 0.202) and N2 and N3 (*P* = 0.188).

### Prognostic value of SUV-T and SUV-N in NPC

**Figure 1:**
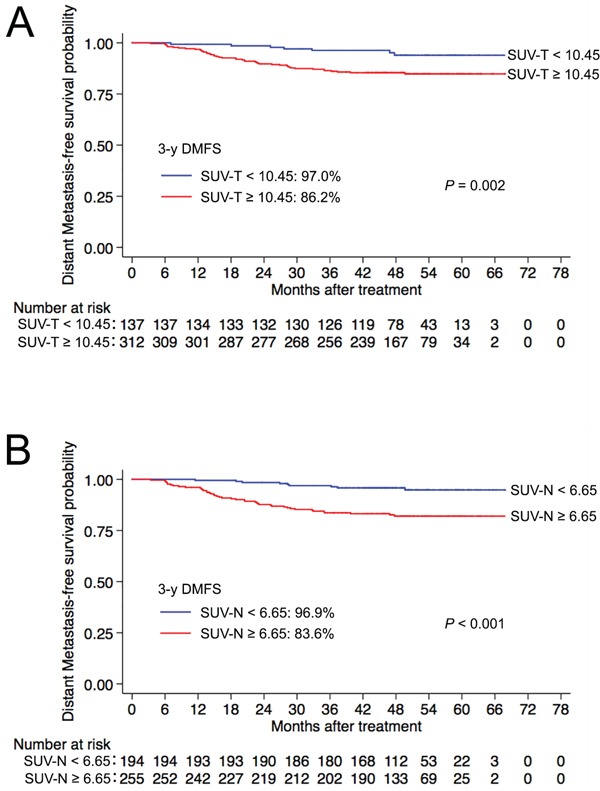
Kaplan-Meier curves of DMFS for nasopharyngeal carcinoma groups **A.** SUV-T and **B.** SUV-N. Abbreviations: SUV-T = SUV_max_ of the primary tumour; SUV-N = SUV_max_ of cervical lymph nodes; 3-y = 3-year; DMFS = distant metastasis-free survival; SUV_max_ = maximum standardized uptake value.

The SUV_max_ for primary tumours ranged from 2.2 to 39.3 (median, 13.6), and the optimal cut-off SUV-T value for distant metastasis was 10.45. This value was selected to classify patients into SUV-T_high_ (≥10.45) and SUV-T_low_ (<10.45) groups. Kaplan-Meier survival curves for the two groups (Figure 1A) showed that 3-year DMFS rates for the SUV-T_high_ group (86.2% vs. 97.0%, *P* = 0.002) were significantly lower than the corresponding rates for the SUV-T_low_ group.

SUV_max_ for cervical lymph nodes ranged from 2.6 to 40.9 (median, 8.4), and the optimal cut-off SUV-N value for predicting distant metastasis was 6.65. This value was selected to classify patients into SUV-N_high_ (≥6.65) and SUV-N_low_ (<6.65) groups. The 3-year DMFS rates for the SUV-N _high_ group (83.6% vs. 96.9%, *P* <0.001) were significantly lower than the corresponding rates for the SUV-N_low_ group (Figure 1B).

Multivariate analysis was performed to adjust for confounding factors. SUV-T (HR, 3.396; 95% CI, 1.451-7.947; *P* = 0.005) and SUV-N (HR, 2.688; 95% CI, 1.250-5.781; *P* = 0.011) were found to be independent prognostic factors for DMFS. Additionally, advanced N-classification (N2-3 vs. N0-1) was also associated with an increased risk of distant metastasis (HR, 2.570; 95%CI, 1.422-4.579; *P* = 0.001).

### RPA-based prognostic model for DMFS

**Figure 2:**
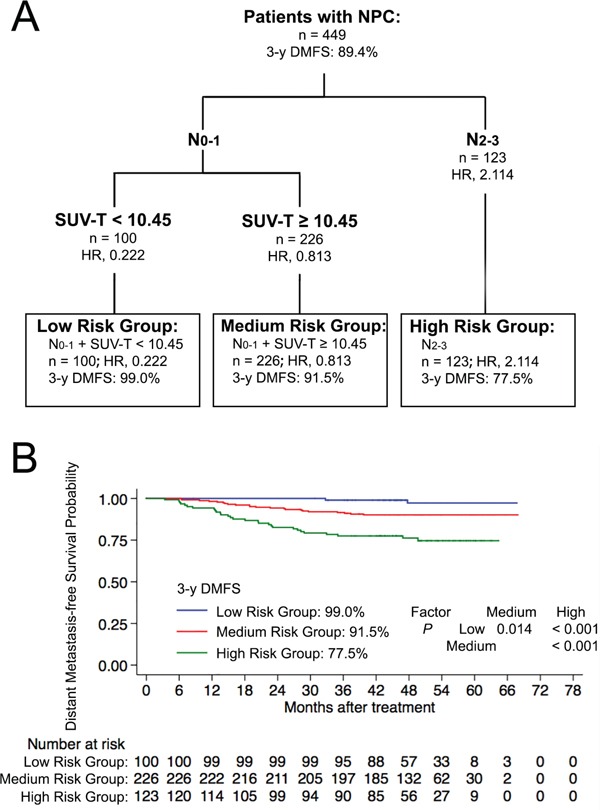
**A.** Prognostic model for DMFS using recursive partitioning analysis (RPA). **B.** Distant metastasis-free survival for derived prognostic groups. Abbreviations: 3-y = 3-year; DMFS = distant metastasis-free survival.

We then used RPA to develop an integrated prognostic model based on the independent prognostic factors identified from multivariate analysis (SUV-T, SUV-N and N-classification). Three valid risk groups were derived: low risk (N0-1 + SUV-T <10.45), medium risk (N0-1 + SUV-T >10.45) and high risk (N2-3). In total, 100 (22.3%), 226 (50.3%), and 123 (27.4%) patients belonged to low, medium and high risk groups, respectively, with corresponding 3-year DMFS rates of 99.0%, 91.5%, and 77.5% (*P* <0.001). Significant differences were observed between the three groups (Figure 2). Multivariate analysis that included host factors (sex, age), tumour factors (T-classification, N-classification), therapeutic intervention (chemotherapy) and RPA-based grouping confirmed the prognostic grouping as the only significant prognostic indicator for DMFS (HR, 3.090; 95% CI, 1.975-4.835; *P* <0.001; Table 1).

**Table 1:** Univariate and multivariate analysis of prognostic factors for DMFS in 449 patients with NPC

Variable	Univariate analysis	Multivariate analysis	
	*P*-value	HR (95% CI)	*P*–value ^a^
Age	0.067	-	0.196
Gender	0.667		0.994
Pathology^b^ (*Keratinizing squamous cell carcinoma vs. Non-keratinizing carcinoma*)	0.484	-	0.968
T^c^ (T1-2 vs. T3-4)	0.033	-	0.155
N^c^ (N0-1 vs. N2-3)	<0.001	-	0.576
RPA group	<0.001	3.090(1.975-4.835)	<0.001
Chemotherapy	0.279	-	0.920

Abbreviations: NPC = nasopharyngeal carcinoma; HR = hazard ratio; CI = confidence interval; T = tumour; N = node; RPA = recursive partitioning analysis.

a *P*-values were calculated using an adjusted Cox proportional hazards model.

b Pathological type according to the 2005 World Health Organization classification of tumours.

c According to the 7th UICC/AJCC staging system.

## DISCUSSION

In this study, we firstly developed an integrated RPA-based prognostic model for DMFS that incorporated SUV-N and N-classification. Using multivariate analysis, the RPA-based prognostic grouping was the only significant indicator for DMFS.

The intensity of tumour FDG uptake is emerging as a valuable predictive factor of treatment outcome [18–20]. ^18^F-FDG uptake, measured by SUV_max_, is correlated with the density and glucose metabolic rate of tumour cells. Tumours with a high pretreatment SUV_max_ are therefore likely to be dense and metabolically active, and are likely to have a poor prognosis [18]. Previous studies reported that the SUV_max_ of primary tumours or regional lymph nodes could predict distant failure in patients with NPC [15, 16], which is in accordance with our results.

Anatomic disease extent reflecting disease burden was the original basis of stage grouping of cancers in the TNM classification [9]. However, more and more non-anatomic prognostic factors are emerging [21, 22]. Even though the UICC and AJCC have recognized that prognostic classifications should extend beyond anatomic parameters alone, a method incorporating non-anatomic prognostic factors that meets the needs of practitioners and researchers has not been reported. Incorporating selected non-anatomic factors into the anatomic classification system while maintaining the consistency and sustainability of the TNM framework is perhaps the biggest challenge.

An RPA-based prognostic grouping incorporating anatomic staging, age, and smoking pack-years for human papilloma virus–related oropharyngeal carcinomas has been recently reported, and this has significantly improved survival prediction [17]. In the present study, we have extended this system by integrating an RPA-based prognostic algorithm with SUV-T and N-classification for predicting distant metastasis. The resultant model identified three distinct risk groups: low risk (N0-1 disease + SUV-T <10.45), medium risk (N0-1 disease + SUV-T >10.45), and high risk (N2-3 disease). This RPA-based prognostic model generated a more balanced distribution and offered superior hazard discrimination compared to N-classification alone, and was confirmed to be the only significant prognostic indicator for DMFS in multivariate analysis.

Despite the promising results, our study has some limitations. Firstly, the proposed model is derived from retrospective analysis of existing data from one institution, and a multi-institution study is needed to confirm our results. Secondly, pretreatment EBV DNA load has been demonstrated to be a valuable prognostic factor in NPC, but this data was only available for a few patients in our cohort and could not be incorporated in our model. Further studies are therefore needed to investigate whether adding EBV DNA data could further improve prediction of metastasis.

In conclusion, analysis of data from a large cohort of NPC patients allowed us to develop an integrated RPA-based prognostic model that incorporates SUV-N and N-classification. Our model performed better at predicting the likelihood of metastasis than previously reported models, and may prove useful for predicting distant metastasis and aiding treatment decisions in the clinic.

## PATIENTS AND METHODS

### Patients

This study was approved by the institutional review board, and the requirement to obtain informed consent was waived. From January 2010 to February 2012, 449 patients with stage I-IVB NPC treated at our institution received a positron emission tomography-computed tomography (PET-CT) examination before treatment followed by intensity-modulated radiotherapy (IMRT) with or without chemotherapy. All of the enrolled patients were of Chinese ethnicity. The median age was 46 years (range, 20- 77), with a male-to-female ratio of 3:1 (Table 2).

**Table 2:** Clinicopathological characteristics of 449 patients with NPC

Characteristics	No. of 449 patients
**Sex**	
*Male*	338 (75.3%)
*Female*	111 (24.7%)
**Age (years)**	
*<50*	302 (67.3%)
*≥50*	147 (32.7%)
**Histological type^a^**	
*Keratinizing squamous cell carcinoma*	4 (0.9%)
*Non-keratinizing carcinoma*	445 (99.1%)
**Chemotherapy**	
*Yes*	385 (85.7%)
*No*	64 (14.3%)
**T-category^b^**	
*T1*	76 (16.9%)
*T2*	76 (16.9%)
*T3*	227 (50.6%)
*T4*	70 (15.6%)
**N-category^b^**	
*N0*	75 (16.7%)
*N1*	251 (55.9%)
*N2*	76 (16.9%)
*N3*	47 (10.5%)
**Stage^b^**	
*I*	22 (4.9%)
*II*	95 (21.2%)
*III*	223 (49.7%)
*IV*	109 (24.3%)

Abbreviations: NPC = nasopharyngeal carcinoma; T = tumour; N = node.

a Pathological type according to the 2005 World Health Organization classification of tumours.

b According to the 7th edition of the UICC/AJCC staging system.

All patients underwent a pretreatment evaluation that included a complete patient history, physical examination, haematology and biochemistry profiles, MRI of the neck and nasopharynx, and PET-CT. All patients were staged according to the 7th edition of the International Union against Cancer/American Joint Committee on Cancer (UICC/AJCC) system [9].

### PET/CT imaging

Serum glucose levels were measured in all NPC patients, all of whom fasted for at least 6 h before PET/CT scans, and individuals with a fasting plasma glucose >200 mg/dl were excluded. PET/CT imaging was performed with a combination PET/CT scanner (Discovery ST 16; GE Healthcare, Little Chalfont, UK) according to published guidelines [23]. Helical CT was performed from the head to the proximal thigh before PET acquisition, according to a standardized protocol and were 45-60 min after injection of 5.55 MBq/kg FDG. PET images were reconstructed from CT data for attenuation correction using an ordered-subset expectation maximization iterative reconstruction algorithm. SUV_max_ was determined for each region of interest using the whole-body attenuation corrected image and the following formula: SUV_max_ = tissue concentration of ^18^F-FDG / injected dose / body weight.

### Treatment

The nasopharyngeal and neck tumour volumes of all patients were treated using radical radiotherapy based on IMRT for the entire treatment course. Institutional guidelines recommended radiotherapy only for stage I and concurrent chemoradiotherapy ± neoadjuvant/adjuvant chemotherapy for stage II-IVB. In total, 92.8% (308/332) of patients with stage III-IVB disease received concurrent chemoradiotherapy ± neoadjuvant/adjuvant chemotherapy. When possible, salvage treatments (intracavitary brachytherapy, surgery or chemotherapy) were provided and persistent disease or relapse was documented.

### Follow-up

Patients were examined at least every three months during the first two years, and every six months during years 3-5 or until death. Evaluation during follow-up included a complete patient history, physical examination, haematology and biochemistry profiles, MRI of the neck and nasopharynx, chest radiography, abdominal sonography and a whole-body bone scan. Any residual disease found at the nasopharynx or cervical nodes within 6 months after completion of RT was regarded as local failure or regional failure, respectively. All distant metastases were diagnosed by clinical symptoms, physical examination, and imaging methods that included chest radiography, bone scan, MRI, CT, PET-CT, and abdominal sonography [24].

### Statistical analysis

Statistical analysis was performed using SPSS version 22.0 (IBM Corporation, Armonk, NY, USA), and distant metastasis-free survival (DMFS) was the defined outcome and was calculated from the first day of treatment to the first distant metastasis. The area under the receiver-operating characteristic (ROC) curve was used to select the optimal cut-off point for SUV-T and SUV-N by maximizing the conditional Youden score, based on the method described by Hanley [25] and Zweig [26]. Survival rates were calculated using the Kaplan-Meier method and compared using the log-rank test [27]. Multivariate analysis based on the Cox proportional hazards model was used to calculate HRs and 95% confidence intervals (CIs), and to test the independent significance of different factors by backward elimination of insignificant variables [28] including host factors (sex, age), tumour factors (T-classification, N-classification), and therapeutic intervention (chemotherapy) as covariates.

Finally, we performed RPA for DMFS to derive prognostic groups that combined anatomic category with other survival predictors identified from multivariate analysis. The RPA algorithm is based on the optimized binary partition of predictors. The resultant subgroups were similar in terms of survival. All tests were two-sided, and *P* <0.05 was considered statistically significant.
